# The AI cycle of health inequity and digital ageism: mitigating biases through the EU regulatory framework on medical devices

**DOI:** 10.1093/jlb/lsad031

**Published:** 2023-12-07

**Authors:** Hannah van Kolfschooten

**Affiliations:** Law Centre for Health and Life, University of Amsterdam, Amsterdam, Netherlands; Amsterdam Institute for Global Health and Development, Amsterdam, Netherlands

**Keywords:** ageism, artificial intelligence, bias, discrimination, EU regulation, medical devices

## Abstract

The use of Artificial Intelligence (AI) medical devices is rapidly growing. Although AI may benefit the quality and safety of healthcare for older adults, it simultaneously introduces new ethical and legal issues. Many AI medical devices exhibit age-related biases. The first part of this paper explains how ‘digital ageism’ is produced throughout the entire lifecycle of medical AI and may lead to health inequity for older people: systemic, avoidable differences in the health status of different population groups. This paper takes digital ageism as a use case to show the potential inequitable effects of AI, conceptualized as the ‘AI cycle of health inequity’. The second part of this paper explores how the European Union (EU) regulatory framework addresses the issue of digital ageism. It argues that the negative effects of age-related bias in AI medical devices are insufficiently recognized within the regulatory framework of the EU Medical Devices Regulation and the new AI Act. It concludes that while the EU framework does address some of the key issues related to *technical* biases in AI medical devices by stipulating rules for performance and data quality, it does not account for *contextual* biases, therefore neglecting part of the AI cycle of health inequity.

## I. INTRODUCTION

*Is there enough information on the safety of medicines for older patients, when clinical trials generally exclude patients above 65 years old? Does face recognition technology still function when people develop facial wrinkles? Will it be possible in the future to access medical records without internet access? Will health professionals offer highly recommended Artificial Intelligence (AI)-based treatment to patients they assume are too old to understand technology? Empirical research shows how chronological age as a sole factor directly impacts the quality of healthcare and overall health status. In healthcare, age is not just a number.*

Europe’s population is aging, and at the same time, there is a growing shortage of health personnel. In response, the use of Artificial Intelligence (AI) medical devices in healthcare in the European Union (EU) is quickly growing.^1^ As life expectancy is improving and healthcare utilization increases with age, the average users of AI medical devices will predominantly be patients of older age, particularly as this group of the population is the main user of healthcare in general.^2^ What distinguishes AI medical devices from classical software medical devices is their ability to autonomously recognize patterns in big datasets and make predictions for individual patients. Many AI medical devices also make use of machine learning techniques and have the capacity to automatically evolve over time based on data input and performance assessment. Examples of AI medical devices that are currently on the EU market include software to automatically detect pulmonary nodules on chest CT scans and track their growth over time, smart hearing aids, and autonomous ophthalmology cameras to detect eye diseases.^3^ Medical AI shows promising prospects for increasing the efficiency, efficacy, and quality of medical care—but not for everyone. Its health outcomes often vary between population groups, including age groups.^4^ While AI can indeed be beneficial for personalizing healthcare for older patients, the risks for precisely this group are often overlooked.

Discrimination is one of the greatest risks posed by automated decision-making systems. Regulators worldwide are racking their brains over how to protect citizens against the harms of biased AI systems without hindering innovation. The ethical AI discourse is mainly focused on gender and racial biases and their risks for discrimination. Pervasive *age*-related biases in AI systems leading to *ageism* go however largely unnoticed and unchallenged.^5^ The lack of scholarly attention to the perception of chronological age in AI is surprising, given that ageism is the most prevalent type of discrimination according to the Eurobarometer on discrimination in the EU.^6^ Butler first coined the term ‘ageism’ in the 1960s, referring to biases, stereotypes, negative attitudes, and discrimination toward older people based upon chronological age.^7^ The terms ‘digital ageism’^8^ and ‘AI ageism’^9^ are used to describe age-related biases in new technology such as AI.

The World Health Organization (WHO) is sounding the alarm about the increasing practice of ageism in *healthcare* in general, and in *medical AI systems* in particular.^10^ Ageism persists especially across healthcare settings, where older adults are commonly stereotyped as physically weak, incompetent, dependent, incapable of autonomous decision-making, or indispensable.^11^ The way in which older people experience discrimination in healthcare is also influenced by intersectional factors such as race, gender, and ethnic origin.^12^ Along the same lines, many AI devices used in healthcare show a correlation between the chronological age of the user and health outcomes.^13^

To explain this phenomenon, this paper introduces the concept of the *AI cycle of health inequity:* existing practices of discrimination in healthcare are programmed into AI systems that replicate these biases in their output, creating a reinforcing loop resulting in health inequity—avoidable—and therefore unfair—systematic differences in the health status of different population groups.^14^ AI systems can generate biases in all phases of the AI lifecycle from data collection to modeling, to application in clinical practice. Biases can be both *technical*, for example when the training data neglect atypical presentation of disease in older adults,^15^ and *contextual*, for example when medical treatment requires the use of a mobile device and digital literacy of older patients is not considered in the deployment of the AI tool.^16^ As a result, AI medical devices are at risk of producing discriminatory results for older patients, posing potential risks to their health and fundamental rights protection. This issue is even more pressing now the average age of AI medical device users is quickly rising. Age discrimination is prohibited under EU antidiscrimination law. But what if the use of AI medical devices causes discrimination?

The EU has obtained an important position in the promotion and protection of nondiscrimination rights and (health) equity, resulting in a broad range of legislative and policy instruments on equal treatment and nondiscrimination.^17^ Consequently, people in the EU have a right to be protected against discrimination. At the same time, the EU has the obligation to take measures to protect the functioning of the internal market. The goal is to ensure the free movement of goods and guarantee high safety standards for consumers. For medical devices, this means that the EU sets legal safety requirements to enter the market under the EU Medical Devices Regulation (MDR).^18^ Manufacturers need to obtain certification for their products and prove the efficacy, quality, and safety of their AI medical devices.^19^ In response to societal concerns about fundamental rights violations, the European Commission has proposed new legislation to regulate AI systems, introducing minimum standards for AI systems in a horizontal AI Act.^20^ This AI Act essentially takes the same product safety approach as the MDR, but also explicitly aims to protect against AI discrimination.^21^ In the case of AI medical devices, the minimum standards proposed in the AI Act create an additional layer to the existing safety and quality standards under the EU MDR.^22^ This multilayered system of regulation for AI medical devices aims to protect both the safety, health, and fundamental rights of users—and at the same time foster innovation. However, while these aims are laudable and harmonization in this field is commendable, it remains to be seen how Member States implement these technical requirements in practice.

The issue of ageism in medical AI plays a role in all layers of EU legislation: it affects fundamental rights (namely nondiscrimination and access to healthcare), but is also an issue of internal market law, as the AI market may not always meet the health needs of older patients. Does the EU regulatory framework for medical devices protect users of AI medical devices against age-related biases and resulting discrimination?

The main objective of this paper is to offer an EU legal perspective on digital ageism in the context of AI in medical decision-making. This paper makes three contributions to the existing literature: (i) it problematizes the lack of attention to AI ageism from a medical, ethical, and legal viewpoint, (ii) it conceptualizes the relationship between biases and health discrimination in the ‘AI cycle of health inequity’, and (iii) it provides a thorough legal analysis of the new EU regulatory framework for AI medical devices from the perspective of bias mitigation. While the legal analysis zooms in on age-related biases, most observations are also applicable to the wider issue of biases in AI medical devices. The legal analysis only focusses on the EU regulatory regime for AI medical devices, but its observations and conclusions may be useful for regulators in other parts of the world as well—as many regions are faced with the challenges of aging populations, persisting ageism, and regulatory questions on balancing the risks and benefits of AI medical devices. This paper does not focus on AI that was specifically designed for elderly care—also known as ‘gerontechnology’—but instead investigates general AI medical devices, designed for a broad category of patients irrespective of chronological age. An explicit choice was made to refrain from further defining ‘older patients’ to not contribute to harmful stereotyping.

This paper proceeds as follows. First, Section II briefly discusses the main medical, ethical, and legal concerns of ageism and discrimination in healthcare. Section III explains how ageism manifests in the design and use of AI medical devices, discussing the various sources of age bias against the background of the AI cycle of health inequity. Subsequently, Sections IV and V assess the EU legislative approach to medical AI, specifically the MDR and the AI Act, evaluating the legal protection for older patients experiencing ageism. The identified limitations of the MDR for addressing age-related biases are used to guide the evaluation of the AI Act. Section V concludes that, while the EU legal framework does address the key issues related to *technical* biases in medical AI, it does not account for *contextual* biases, therefore neglecting part of the cycle of health inequity.

## II. AGEISM AND EU NONDISCRIMINATION LAW IN HEALTHCARE

Ageism refers to stereotypes, negative attitudes, and discrimination toward older people based on chronological age.^23^ Ageism persists especially across healthcare settings.^24^ Unconscious (or implicit) age-related biases are widely displayed in both individual behaviors (eg by health professionals) and in systematic barriers (eg in the design of healthcare systems). Several studies on age bias in breast cancer treatment recommendations show how health professionals were less likely to recommend surgery (the gold standard treatment for breast cancer) for older patients as compared with identical younger patients, which illustrates the detrimental impact of age bias in health professionals on health outcomes for older adults.^25^ Health professionals are also often unaware of atypical symptoms of many diseases in older adults risking missed diagnoses.^26^ As for systematic barriers, a comprehensive systematic review carried out by researchers from Yale University comprised data from 7 million older people from 45 countries in 422 studies carried out between 1969 and 2017 showed a strong association between ageism and a wide range of health outcomes globally.^27^ For example, older patients were significantly more likely to be denied access to health services and treatments, and older people were routinely excluded from clinical trials leading to underrepresentation in medical knowledge (eg in clinical trials of Alzheimer’s disease^28^).^29^ The health risks of ageism are exacerbated by other intersectional factors, such as gender (eg older women^30^), race, ethnicity, and socio-economic status. In conclusion: ageism in healthcare results in lower quality healthcare, and lower health outcomes for older adults.^31^

Recently, the WHO called for urgent action to combat ageism in AI technologies because of its risks to the health and well-being of older adults.^32^ The increasing use of AI medical devices, for example in the detection of early signs of breast cancer in mammograms (diagnostics), or clinical decision support systems on breast cancer treatment decisions (treatment recommendations), may reinforce societal patterns of age bias and discrimination. *Bias in AI* refers to the tendency of AI systems to produce consistently different decisions for one group compared with another, resulting in unfair outcomes that show bias toward a select group of individuals, such as older people. Age impacts, for instance, the performance rate of AI medical devices using biometric technology for diagnosis and monitoring (ie facial recognition, voice recognition, and fingerprint scanners).^33^

In addition to posing health risks, the expanding use of AI medical devices raises pressing concerns for the protection of the right to nondiscrimination and the right to effective access to healthcare for older patients. The right to nondiscrimination is a human right: in the context of Union law, Article 21 EU Charter on Fundamental Rights (CFREU) prohibits any discrimination based on any ground, including age. Both *direct* and *indirect* discrimination in healthcare is prohibited under EU antidiscrimination law. With regard to healthcare, the EU has obtained an important position in the protection and promotion of (health) equity. Equity is both an EU value, a general principle of EU law, and an EU fundamental right.^34^ Applying the principle of equity to healthcare, persons in equal need of healthcare should have equal access to it. This is reflected in Article 35 of the CFREU, which guarantees equal access to healthcare in accordance with national legislation.

Chronological age is a protected characteristic under EU nondiscrimination law. In addition, the CFREU protects the rights of the elderly in Article 25, recognizing their rights to lead a life of dignity and independence, and to participate in social and cultural life. In 2000, the EU adopted specific rules to protect against discrimination at work on grounds—amongst others—age.^35^ A framework for the prohibition of (age) discrimination *outside of the labor market* was proposed in 2008.^36^ The EU also explicitly acknowledges the risk of age-related discrimination in its AI strategy and AI Act proposal.^37^

In principle, the EU’s legislation on nondiscrimination and equality creates obligations for protecting against age-based discrimination stemming from biases in AI medical devices. There are however three main difficulties with the EU antidiscrimination framework for combatting ageism in medical AI systems: (i) EU law protects against discrimination in access to healthcare *only* on grounds of sex and racial or ethnic origin, (ii) EU law does not account for intersectional factors leading to multiple discrimination (eg people who experience discrimination because of gender *and* age group), and (iii) the untransparent ‘black box’ nature of AI makes it difficult to prove causality. The EU nondiscrimination framework has been criticized for not accommodating the specific challenges posed by ‘automated discrimination’ by AI systems.^38^

Ethical concerns are expressed in the wider context of health inequity. Health inequity relates to systemic, avoidable differences in the health status of different population groups.^39^ As medical AI is adopted unevenly across age groups, or disproportionately benefits certain age groups, this may widen the digital divide between generations and deepen health inequity. The next section shows how ageism is produced throughout the entire lifecycle of medical AI and creates a vicious cycle of health inequity amongst older people: the AI cycle of health inequity. In this paper, digital ageism is used as a use case to show the inequitable effects AI can have on specific population groups. That said, the AI cycle of health inequity is also applicable to other types of biases.

## III. DIGITAL AGEISM AND THE AI CYCLE OF HEALTH INEQUITY

Ageism is both a health and a fundamental rights issue, and AI medical devices may exacerbate existing challenges for older adults. AI medical devices use algorithms to construct knowledge from large datasets to make medical decisions based on the processing of the patient’s personal data or profile. To do so, AI depends on—potentially biased—large datasets. The way devices are deployed and used can also contribute to ageism.^40^ To understand how medical AI may mimic patterns of ageism and discrimination, this section first identifies the *sources* of age-related bias in medical AI and how this may lead to ageism. This is important to understand the working of the AI cycle of health inequity and to eventually evaluate the role of the law in mitigating ageism produced by medical AI.

**Figure 1:**
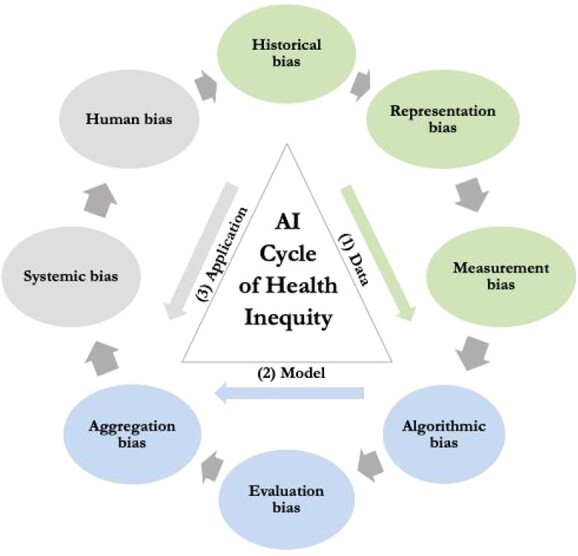
The AI Cycle of Health Inequity

As shown in Figure 1, AI systems can produce and reinforce biases in multiple stages of the AI lifecycle: the (i) *data*, (ii) *modeling*, and (iii) *application* stage, and can be caused by various sources.^41^ The biases in the phases of data and modeling are often referred to as the ‘technical factors’. AI systems function on large datasets, and biases in these datasets may lead to discriminatory outcomes. Biased variables or proxies may also be programmed into the algorithm. However, there is more to bias than biased data, and biases can also arise from the application phase of the AI system, depending on ‘contextual factors’ such as effective access to digital technology, which functions as a key determinant of health.^42^ Health is influenced by factors such as digital literacy, technology access, and health professionals’ perceptions of an individual’s digital literacy,^43^ which may vary by age. The way health technology is designed and the law and policies around it can also be seen as a determinant of health (ie rules on data quality and inclusivity), as certain age groups may be excluded in the design and use phase of medical AI.^44^

 In the data stage, ageist practices in society may be reflected in the dataset that is used to develop the algorithm. First, algorithms may be trained with data that no longer accurately reflect reality, and *historical bias* in society will be mirrored in the output. Historical bias could lead to ageism when, for example, the algorithm was trained on Eurostat data from 2015, where only 24.5 per cent of EU respondents in the age category 65–74 years had reported having used the internet for seeking health information during the 3 months prior to the survey, instead of the 2022 survey, where 36.16 per cent of 65–74-year-olds did so.^45^  *Representation bias* occurs when certain population groups are underrepresented in the training dataset, for example, clinical trial data that often exclude older adults,^46^ and the medical AI trained with data from predominantly younger populations is used for a population of older people. *Measurement bias* exhibits when the training or validation data are inaccurate (eg in case of erroneous input data, such as inaccurate medical records) or when data are labeled incorrectly (eg using the same variables for cardiovascular diseases in women and men^47^).

In the modeling stage, age-related biases may be programmed into the algorithm or model. First, *algorithmic bias* surfaces when a biased variable or proxy is introduced into the model, such as using age as a proxy for treatment preference (eg using chronological age as a proxy for the willingness to undergo breast cancer surgery^48^). *Aggregation bias* is exhibited when a general model is used for groups with different conditional distributions (eg neglecting atypical presentation of disease in older adults^49^), and *evaluation bias* when the data used to test the performance of an algorithm do not represent the target population (eg using the data of patients with preclinical Alzheimer’s disease to test an AI device targeted to patients with late-stage Alzheimer’s disease).^50^

Bias in AI medical devices can arise from other sources than the underlying data. In the application stage, AI systems may produce or reinforce ageism through the way they are deployed or used in society. The context in which AI medical devices are used influences the outcomes, for example when a system developed for a high-resource context is applied in a low-resource context.^51^ For older people living in low-resource environments, this contextual bias could exacerbate existing health disparities. Ageism may be caused by human age-related biases in individual behaviors (eg by health professionals or the government) and in systematic barriers (eg in the design of healthcare systems, or the infrastructure).^52^  *Systemic bias* refers to the tendency for the practices of institutions to operate in a way that advantages certain social groups and disadvantages others, for instance in creating barriers to access to healthcare. For example, requiring patients to enter health data in an online system may disproportionately disadvantage older patients. The digital divide between younger and older populations in using new technologies is growing because of disparities in digital literacy.^53^ In 2022, as much as 35.55 per cent of EU respondents in the age category 65–74 years reported having never used the internet, as compared with 10.37 per cent of all respondents over 16 years.^54^ Another example is the systematic exclusion of older adults from clinical trials, leading to underrepresentation in AI datasets, or rationing of health resources on the basis of chronological age alone.^55^  *Human bias* may surface when people decide how to interpret the outcomes of AI systems or missing data links. Unconscious biases, prejudices, and stereotypes toward older patients play a large role in the creation of human bias, for example not recommending a technological solution to an older patient because of the assumption that the patient does not understand technology. In conclusion: the risks for biased AI medical devices relate to the way data is used in the development *and* use phase of the devices.^56^

The various sources of age-related biases in the lifecycle of AI interact and lead to a vicious ‘AI cycle of health inequity’. Older people are already vulnerable to health inequity because of unconscious age-related biases in health professionals and structural barriers in the health system. Ageist stereotypes in society may lead to the exclusion of older people in clinical trials, which will lead to the underrepresentation of older people in datasets, resulting in lower performance rates for older people, eventually reinforcing existing stereotypes. The adverse effects of AI-mediated ageism are exacerbated by other intersectional social factors.^57^ In light of its strong fundamental rights framework, and recent efforts to regulate AI, the next section examines whether the EU regulatory framework adequately addresses the challenges posed by the AI cycle of health inequity for older adults.

## IV. THE LIMITATIONS OF BIAS MITIGATION IN THE EU MEDICAL DEVICES REGULATION

EU regulators are faced with the complex balancing exercise of protecting citizens against discrimination, ensuring health protection, and protecting the functioning of the internal market. The EU is founded on the values of respect for human dignity, freedom, democracy, equality, the rule of law, and respect for human rights. As such, the EU must protect its citizens against discrimination.^58^ The EU also has a responsibility toward the protection of a high level of human health in all EU activities and policies,^59^ and in this light, can adopt legislation to set high standards of quality and safety for medical devices.^60^ A regulatory framework surrounding medical devices—as goods on the EU internal market—is also an important task of the EU as guardian of the functioning of the internal market.^61^ These various interests come together in the questions of regulation of AI medical devices. This leads to a fragmented, multilayered system of EU regulation of AI medical devices, with the AI Act proposal as the most recent layer. This section first discusses the applicability of the Medical Devices Regulation (EU) 2017/745 (MDR), which was issued in May 2017 and entered into force in May 2022. Subsequently, it evaluates how this framework responds to the challenges of bias and ageism posed by AI medical devices. The next section then evaluates the contribution of the AI Act proposal for bias mitigation.

### IV.A. AI Medical Devices under the MDR

The MDR, the main regulatory instrument for medical devices in the EU, aims to ‘establish a robust, transparent, predictable and sustainable regulatory framework for medical devices which ensures a high level of safety and health whilst supporting innovation’.^62^ It regulates the safety and effectiveness of medical devices on the EU market. The EU MDR has a broad scope of application, covering more than 500,000 different types of medical devices in the EU, ranging from bandage plasters and pacemakers to software.

Whether a product qualifies as a ‘medical device’ under the MDR first depends on its intended use: it must be intended by the manufacturer to be used for human beings for specific medical purposes, such as diagnosis, prevention, or treatment of disease.^63^ In light of the MDR’s definition and case law on the previous Medical Devices Directive, it can be concluded that software may be qualified as medical devices under the MDR, depending on the intended use.^64^ This applies to both software to be used alone (*stand-alone software*, ie prescription support software) and in combination with a medical device (*software as medical device*, ie software that calculates required insulin dose, which leads to the insulin pump administering the calculated dose).^65^ In the first instance, the software functions as an *accessory* for a medical device, while in the second instance, the software drives or influences the use of a (hardware) medical device.^66^ Following this, most AI tools used for medical purposes will qualify as medical devices under the MDR.

The MDR establishes four different classes of medical devices, according to their level of risk. The MDR classifies ‘software intended to provide information which is used to take diagnostic or therapeutical purposes’ and ‘software intended to monitor physiological processes’ as Class IIa, and as Class IIb if it is intended for monitoring of vital physiological parameters (ie heart rate), in which case it is classified as Class IIb. If the decisions flowing from the AI medical device’s recommendation on treatment or diagnosis have an impact that may cause death or an irreversible deterioration of a person’s state of health, it is classified as the highest risk class: Class III. All other software is classified as Class I.^67^ This means that most AI medical devices will be classified as at least Class IIa or IIb, and thus are subject to a stricter regulatory regime. To enter the EU market, the AI medical device is subject to a conformity assessment leading to certification (CE marking) by notified (private) bodies.^68^ The medical device manufacturers must demonstrate compliance with the General Safety and Performance Requirements listed in the MDR.^69^ This assessment involves a review of the manufacturer’s provided technical documentation on the safety and clinical performance of the medical device, and its risk assessment system. For implantable devices and for class III devices, a summary of the safety and clinical performance of approved medical devices is published in the publicly accessible EUDAMED database, coordinated by the European Commission.^70^ Manufacturers must also report serious incidents in the database.^71^

### IV.B. Bias Mitigation in AI Medical Devices Assessment

How does the MDR mitigate harmful biases for older patients? The MDR addresses bias as a potential performance issue—as a threat to the quality and safety of medical devices. In theory, the requirements of the MDR for AI medical devices, and especially the required clinical evaluation and assessment by notified bodies, may reveal biases for specific age groups that would otherwise have gone unnoticed. Manufacturers are also required to set out in their clinical investigation plan the details of measures to be taken to minimize bias, such as randomization, and management of potential confounding factors.^72^ Apart from the requirements on safety and clinical performance, the MDR requires manufacturers to monitor the post-market performance of their devices, including any potential biases that may arise over time as new data are collected. Finally, the MDR requires manufacturers to provide transparency and information to users about their devices in the EUDAMED database. Combined, the measures required by the MDR could help to limit biases in the training data (data phase) and in the algorithm itself (model phase). However, the MDR does not specifically mention AI medical devices. The next section discusses the shortcomings of the MDR framework for mitigating (age-related) biases in AI medical devices.

#### IV.B.1. Lack of Specification of Sub-Populations for Clinical Evaluation

To demonstrate the accuracy of the performance of their devices, manufacturers have to conduct a clinical evaluation of their products.^73^ It is however not clear exactly how manufacturers can provide regulators with sound clinical evidence to prove performance and compliance. The MDR broadly lists the requirements. These requirements are then elaborated on in harmonized standards, which can be used to demonstrate compliance.^74^ The clinical evaluation should be conducted in accordance with the intended purpose of the device, for a specified target population, such as adults and/or children and/or infants, since clinical performance may vary between certain population groups.^75^ This means that manufacturers need to look for historical, representation, and measurement biases in the training dataset, in relation to the target population, to evaluate differences in outcomes for sub-populations in the target group.^76^ Alongside age, racial and gender biases are important factors to consider. This is where the first limitation of the MDR for age bias mitigation comes in: it considers patients of adult age to be a homogenous group and does not require clinical evaluation for specific age groups, only broadly differentiating between adults, children, and infants. If the medical device is targeted toward a general patient population, the MDR does not specify how many sub-populations need to be studied to make sure the device contains no age biases.

#### IV.B.2. Limited Guidance and Transparency on ‘Ground Truth’ Selection

The second limitation relates to the ‘ground truth’ to train algorithms and later evaluate the AI’s performance. The ‘ground truth’ is the information that is known to be true—the independent reference standard reflecting the correct answer to a specific question.^77^ This is often established by experts in the field, such as radiologists. A high-performing AI medical device should match the ground truth, for example, detecting a long nodule in a chest X-ray that experienced radiologists labeled as a ‘positive detection’ of this lung nodule. To evaluate its accuracy, the results flowing from the AI medical device need to be checked against this ground truth.^78^ However, developers of medical devices use various reference databases to evaluate performance. These reference databases may also contain age-related biases, for example stemming from human biases in the labeling by radiologists, or from representation biases in clinical trial data because of upper age limits.^79^ The MDR does not require disclosure of the exact ground truth that was used and what clinical guidelines were used to establish this ground truth. This is another example of measurement bias that may be programmed into the algorithm, and evaluation bias surfacing from using a biased ground truth dataset.

#### IV.B.3. Uncertain Interaction with Data Protection Laws

Another limitation is the lack of guidance the MDR provides on the interaction of the regulation of AI medical devices and data protection. Manufacturers of AI medical devices may be reluctant to share extensive information on the algorithm and dataset of their devices with the notified bodies. This may be because of competitive reasons, but also because of the sensitive nature of the data and prohibitions under the General Data Protection Regulation (GDPR).^80^ The MDR requires to share sensitive information about the clinical evaluation, but at the same time requires manufacturers to protect the confidentiality of personal data and health data of research participants.^81^ The MDR does not provide further clarification on how exactly the regulatory requirements interact with the GDPR. As a result, it may be difficult for the notified bodies to review the performance of the AI medical device for a specific age group—as they may lack access to important patient data.

#### IV.B.4. Limited Transparency and Public Disclosure

In relation to potential problems with transparency in the conformity assessment phase, another limitation may be the limited transparency on health outcomes for specific (older) age groups warranted by the MDR. The MDR only requires manufacturers of implantable and Class III medical devices to summarize ‘the main safety and performance aspects of the device and the outcome of the clinical evaluation’ in the publicly accessible EUDAMED database.^82^ This means that for most AI medical devices, this information is not available. As a result, there is limited public disclosure of performance on subpopulation-specific data. This lack of transparency makes it impossible to inform patients or health professionals about whether new medical devices are safe and effective for specific age groups and to include this as a consideration in medical decision-making for individual older patients.^83^ Another challenge is that the EUDAMED database currently does not specify whether the approved medical device makes use of AI techniques—it only specifies whether it is software.

#### IV.B.5. Latent Biases and the Update Problem

AI medical devices present a new problem to regulators: the so-called ‘update problem’.^84^ Many systems make use of algorithms that develop over time, learning from and adapting to new situations. This means that the AI medical device that was granted a CE marking will not necessarily perform in the same manner over time and could thus exhibit unforeseen age-related biases after they are deployed in clinical practice, also referred to as ‘latent biases’.^85^ For example, an algorithm predicting individual patients’ responses to specific treatments could learn from existing healthcare disparities—for example age-related—and predict worse outcomes for older patients. Latent biases can also surface in the application phase of the AI medical device, for example when the device is applied in a low-resource clinical context.

Under the MDR, manufacturers must inform the notified bodies of changes in the device that could affect the safety and quality of the product. It can be argued that this is also the case for adaptive algorithms producing significantly different—harmful—output. In this case, the notified body can decide to start a new conformity assessment or add a supplement to the CE certificate.^86^ This practice may be problematic for bias mitigation, as it can take a long time for manufacturers to update their AI software according to newly available research in the field, or after a harmful bias was discovered.^87^ For example, it could become clear only in a later stage that the algorithm was trained on data that did not distinguish between heart attack symptoms in younger and older adults, therefore providing incorrect diagnoses for older adults with heart disease.

#### IV.B.6. Lack of Technical and Human Capacity

A central challenge is the lack of understanding of the sources of bias in AI medical devices, both from the side of the manufacturer and notified bodies. The MDR does not provide extra guidance in mitigating this knowledge gap. In practice, it is questionable whether the notified bodies are equipped to review large datasets and the—often opaque—algorithms. For AI medical devices, notified bodies would need to review both the algorithm and (a description of) a large training/input dataset to verify compliance with the safety and performance requirements.^88^ This requires extensive human capacity. In 2022, EU health ministers expressed their concerns about the limited capacity of notified bodies. Another issue may be the technical nature of an algorithmic assessment.

#### IV.B.7. Lack of Guidance on Bias Assessment

In addition, there is limited guidance on how exactly the notified bodies can assess the equitable performance of AI medical devices. The requirements laid down in the MDR are rather abstract and are not specified for AI devices. While the European Commission’s expert committee on medical devices (MDCG)^89^ published guidance on the assessment of software medical devices, it does not specifically advise on the challenges AI software brings along.^90^ Both the European Commission, responsible for scientific, technical, and logistical support to national authorities in the field of medical devices,^91^ and the EMA (the EU body for regulation of pharmaceutical products) currently leave the issue with the individual notified bodies and the EU Member States. This means that notified bodies decide for themselves how to evaluate for biases in AI medical devices, also opening the door to ‘forum shopping’ for the notified body with the most lenient procedure regarding clinical data review, with potentially harmful effects for specific patient populations. Private actors such as medical associations or groups of academics have been trying to fill this gap by publishing guidance documents, for example on how to perform bias assessment in prediction models, and checklists for evaluation of AI-generated medical reports.^92^ Global standard organizations such as ISO and IEC also issue standards and guidelines for medical devices, although as of today, specific standards on AI medical devices are missing. The expectation is that these private organizations will propose guidelines for quality assessment of AI medical devices in the future, including guidance on bias assessment.^93^ Because of the private governance system of medical devices in the EU, it seems however unlikely that either the European Commission or the EMA will publish a regulatory approach to clinical evaluation of AI medical devices, similar to the centralized US Food and Drug Administration (FDA) that recently published a (draft) guidance and action plan on oversight of AI medical devices.^94^

#### IV.B.8. Neglecting the Human Factor

A final limitation is that the MDR insufficiently considers the human factor: how the medical device will be used by the health professional and patient in the clinical practice. It thus only addresses part of the AI cycle of health inequity: the data collection phase and possibly the modeling phase. It is focused on ensuring the functioning of the internal market and setting high standards for the quality and safety of products, protecting the health of users. It does not aim to regulate the ‘user phase’ of medical devices, where health professionals and patients must make medical decisions on the use of a specific medical device for a specific patient. This can be harmful to older patients since digital ageism arises from more sources than only the data that were used. Health professionals may prescribe medical devices that are unsuitable for the individual patient or may refrain from providing the patient with adequate information on the proper use of the device. In this way, ageist human biases may creep into the medical device. At the same time, it is known that EU Member States have a large degree of freedom in shaping their healthcare systems and policies, and the EU’s power to legislate is limited.^95^

In conclusion, the MDR theoretically provides some protection against biases arising from AI medical devices for older patients from a quality and safety perspective by focusing on data quality and introducing a market oversight regime. It is however questionable whether the MDR provides adequate legal tools for ageism detection in AI medical devices and to what extent the notified bodies are up for this task. Plus, biases and discrimination—specifically digital ageism—cannot be solved by addressing data quality alone.

The MDR is clearly not a nondiscrimination instrument and does not account for contextual biases surfacing in the application phase of medical AI. It is therefore necessary to look into other potential EU-level legislation that seeks to address these particular shortcomings under the MDR. The EU proposal for an AI Act, while technically also a product safety instrument, does aim to protect fundamental rights—including nondiscrimination. The next section explores the potential advantages of this new framework for mitigating age-related biases in AI medical devices.

## V. SOLUTIONS FOR BIAS MITIGATION IN AI MEDICAL DEVICES IN THE AI ACT?

In April 2021, the European Commission proposed the first horizontal framework for AI regulation: the EU AI Act. The European Commission has opted to regulate AI systems as ‘products’ (as opposed to ‘services’) in line with the ‘New Legislative Framework’ (NLF)—a package of EU legislation on product legislation covering a wide range of products with the aim of improving the internal market for goods. The main idea behind the NLF is that the manufacturer of a product is responsible for its conformity with applicable rules and must therefore prove its conformity—in some cases to be evaluated by independent third-party authorities—before they can enter the EU market. In this light, the AI Act follows the same regulatory regime as the MDR.

However, within the NLF framework, the AI Act proposal occupies a special position between product safety regulation and fundamental rights protection.^96^ One of the main aims of the AI Act is to establish ‘a high level of protection of health, safety, and fundamental rights’.^97^ The AI Act proposal acknowledges the large risks of discriminatory AI medical devices. While the AI Act proposal has a general scope, it also stipulates rules for medical devices as high-risk AI applications.^98^ One could argue that in this sense, the AI Act will add a new ‘fundamental rights’ layer to the AI medical devices covered by the MDR. On the other hand, the AI Act is—like the MDR—a product safety framework and introduces a parallel pre-market authorization regime for AI medical devices.^99^ What is the interplay of this new regime with the current regulatory system for AI medical devices and how do these respond to the risks of AI medical devices for ageism? This section evaluates the text of the legislative proposal as put forward by the European Commission on April 21, 2021. It is important to note that the current text may face substantial changes due to the design of the legislative procedure within the EU, where the adoption of legislative proposals is dependent on approval by the Council and the European Parliament.^100^ The proposed provisions are currently heavily debated amongst the Commission, Council, and Parliament in the ‘trilogue’-phase.

### V.A. AI Medical Devices under the AI Act Proposal

The AI Act classifies AI medical devices as high-risk systems when they are (i) intended to be used as a safety component of a product or an independent product, (ii) covered by the MDR, and (iii) require third-party conformity assessment.^101^ In practice, this means that all AI medical devices classified as Class IIa or higher under the MDR must comply with the rules for high-risk AI systems stipulated in the AI Act. As a result, the current conformity assessment of the MDR will most likely be complemented by the new requirements arising from the AI Act. Manufacturers of AI medical devices also need to register their devices in the proposed EU AI database.

At the same time, however, the AI Act proposal is a general framework and applies to a broad spectrum of AI systems and sectors outside of healthcare and may thus not take into account health-specific considerations.^102^ A scan of the documents making up the EU approach to AI regulation also shows that age-related biases and AI ageism in healthcare are not on the EU agenda.^103^ In general, ‘healthcare’ is only mentioned once in the current AI Act proposal.^104^ The particular risks AI poses to older people are not specifically considered, while there is attention to the vulnerability of people of younger age and children.^105^ Risks specific to older persons such as digital literacy and the ‘digital divide’ are not mentioned in the proposal.^106^ The AI Act proposal does specifically mention risks of AI systems that exploit ‘any of the vulnerabilities of a specific group of persons due to their age’,^107^ acknowledges that age can be a factor of vulnerability,^108^ mentions the risks for age in AI remote biometric identification systems,^109^ and highlights the risks of AI for age discrimination on the work floor and credit scoring.^110^ Unlike other EU fundamental rights instruments,^111^ the AI Act does not introduce individual rights for end-users of AI products, such as patients using AI medical devices. Its provisions can however indirectly contribute to the fundamental rights protection of patients.

### V.B. Bias Mitigation in AI System Assessment

Under the AI Act proposal, AI medical devices must undergo pre-market conformity assessments. Before entering the EU market, providers must demonstrate compliance with the requirements under the AI Act. For the assessment of AI medical devices, the AI Act envisages a key role for the current notified bodies under the MDR. The AI Act proposes three main categories of requirements to mitigate the risks of AI systems for discrimination: data quality requirements,^112^ transparency requirements,^113^ and human oversight requirements.^114^ While these requirements complement the MDR and address its current shortcomings for bias mitigation in various manners, the AI Act proposal leaves certain issues uncovered. It is important to note that the European Commission is simultaneously working on the establishment of a ‘European Health Data Space’ with the aim of creating a common space with high-quality health data that can also be used for the development of high-quality medical devices.^115^

#### V.B.1. Data Quality Requirements

The AI Act proposal attaches high importance to high-quality data for the performance of AI systems and for reducing the risks of discrimination. Article 10 sets quality criteria for datasets of high-risk AI systems—including AI medical devices—with the aim of mitigating biases in datasets. It stipulates that ‘training, validation, and testing data sets shall be relevant, representative, free of errors, and complete.’ The quality must be assessed with regard to ‘the persons or groups of persons on which the high-risk AI system is intended to be used’, and the choice of dataset shall also take into account ‘characteristics or elements that are particular to the specific geographical, behavioural or functional setting’ of the intended use.^116^ Providers of AI systems are also required to have systems in place for bias monitoring, detection, and correction.^117^ The data quality requirements address the various biases that are prone to occur in the data stage (ie representation bias in training data) and modeling stage (ie evaluation bias in the testing dataset) by stipulating ex ante obligations in the developing, pre-marketing stages. It also urges developers to consider the specific context in which the AI system will be used, which diminishes the risk of aggregation bias. In general, obligating developers of AI systems to run extra tests to evaluate for biases may in fact lead to a decrease in biased AI medical devices. It also puts more emphasis on the role of the notified bodies in bias assessment. The data quality requirements are further strengthened by the obligations of record-keeping and the requirement to provide a regulatory body with technical documentation before entering the market.^118^

While the requirements address many potential sources of bias, especially those in the data and modeling phase, the AI Act proposal is limited to biases in the underlying data. Contextual biases related to stereotypes, prejudices, and (un)conscious biases are not regulated. This shows how the AI Act functions as a product safety instrument rather than a fundamental rights instrument, in spite of its ambitious objective to bridge both aims.^119^ In the absence of provisions relating to biases in the application phase, such as harmful age-related stereotypes, ageist biases in medical decision-making by health professionals and differing levels of digital literacy between age groups, an important part of the AI cycle of health inequity remains unregulated. Another flaw in the data quality requirements for the purpose of bias mitigation may be the lack of specification of relevant characteristics vulnerable to the risks of bias, such as age. This is problematic for combatting digital ageism because while some biases such as sex, gender, and race are now on the radar of regulators, age-related bias in AI medical devices is a rather invisible issue of discrimination.^120^

A caveat is that the data quality requirements are dependent on the *intended use* set by the developer. This could mean that the developer defines the intended use very narrowly (ie a software prediction system to assess whether Covid-19 patients need intubation is intended to be used only in high-resource hospitals for white, male patients between 45 and 65 years old)—excluding a lot of patients from accessing this type of care. It could also mean that, in practice, the responsibility of evaluating the safety of a specific AI medical device for an individual patient falls on the health professional using or prescribing the device, thus shifting the burden of risk. At the same time, while the AI Act’s data quality requirements outline in more detail the diversity of data that is needed to develop AI medical devices, it does not provide AI medical device developers and notified bodies with more guidance on how specified the sub-populations must be, how to perform bias assessments, and how to identify and select a reliable ground truth. This is left to the self-assessment by the AI developers and providers, and then to the conformity assessment performed by regulatory bodies—which brings us back to the previously discussed shortcomings of the MDR framework.

#### V.B.2. Transparency Requirements

Transparency of AI systems is a key ethical and legal requirement underpinning the AI Act proposal. Transparency requirements are also an important part of the solutions the AI Act provides to mitigate the risks of biased AI systems producing discriminatory outcomes. AI providers must demonstrate compliance with the following main transparency requirements: (i) systems must be sufficiently transparent to enable users to understand the system’s output and assess the system’s risks, (ii) there must be sufficient transparency of the functioning of the system to allow for effective human oversight, and (iii) users must be aware that they are interacting with an AI system.^121^ On top of this, the European Commission sets up a public EU database for stand-alone high-risk AI systems, where providers of AI systems have to enter information about their AI systems, including information on potential incidents.^122^

These measures may address some of the shortcomings in the MDR regarding transparency. While the EUDAMED database only requires certain medical devices to publish the main safety and performance aspects of the device, the AI Act extends the obligation for public disclosure of certain information to *all* AI medical devices. This could help both patients and health professionals to make informed decisions. Linking the AI database to the EUDAMED database could also create more clarity on whether the approved medical device makes use of AI techniques. The AI Act however also does not require public disclosure of the details of the clinical evaluation—including bias mitigation measures—therefore still not allowing health professionals and users to evaluate the effects of the use of AI medical devices for specific age groups by consulting the database. That said, this risk may be covered by the extensive transparency requirements in Article 13, stipulating strict rules as to the information to be shared with the user of the device in the accompanying instructions (in the case of AI medical devices: the health professional), including information on potential biases and performance for specific groups.

However, as also the AI Act largely depends on the intended use by the manufacturer of the AI system, the transparency obligations also must be evaluated in relation to the intended use. That means that, if the AI medical device is intended to be used by a general patient population, the AI Act—like the MDR—does not provide further guidance on specified sub-populations such as qualified age groups.^123^

#### V.B.3. Regulatory Oversight Requirements

The AI Act proposal introduces a new pre-market authorization regime for AI systems. In the case of AI medical devices, it is envisaged to ‘melt’ the new AI requirements with the existing product safety framework of the MDR and expand the authority of the notified bodies currently regulating medical devices with the new AI regulatory regime. The AI Act proposal is just one of the links in a chain of product regulation: it adds an extra layer to the MDR for medical devices using AI. While it is useful that the AI regulatory framework builds on the existing expertise of medical device regulators, instead of introducing an entirely new regulatory body, this solution does not address the burden of regulation.^124^ It does not solve the lack of technical and human capacity of notified bodies—in fact, it only adds more tasks to the regulatory bodies. It remains to be seen whether the designation of regulatory oversight to the notified bodies will indeed reduce biases and discrimination stemming from AI medical devices.

In theory, the accompanying system of market surveillance introduced by the AI Act can be an important tool in mitigating the risks of bias and ageism, as the AI cycle of health inequity shows that bias can emerge in different stages of the AI lifecycle. In practice, however, the AI Act does not seem to provide the legal and technical tools to assess the surfacing of contextual biases in the use of AI medical devices for individual patients. This can be explained by the NLF approach to the AI Act, where, in the end, the regulatory focus lies on the safety and quality characteristics of the product *itself*. The special challenge of AI devices lies however in how the product interacts with the external world throughout its whole lifecycle, from the developmental phase to the input of an individual’s personal data into the system, to its effects on society. By regulating AI as a product instead of a system, the EU neglects the *human* factor of AI.^125^ That said, the AI Act does address the ‘update problem’, by allowing providers of AI systems to report ‘predetermined changes’ that arise from adaptive algorithms in the initial conformity assessment.^126^ In these instances, a new conformity assessment is not necessary, and manufacturers of AI medical devices can correct harmful biases in a timely manner.^127^

Bias monitoring by the provider is one of the requirements for AI providers. The AI Act stipulates that to ‘debias’ AI systems and prevent discrimination, it may be necessary to process special categories of personal data, therefore introducing a new exemption from the prohibition stipulated in Article 9 GDPR to process sensitive data.^128^ Providers are required to implement appropriate safeguards such as anonymization or pseudonymization. While generally age is not protected as a special category of personal data under the GDPR, health data are, and age can in specific cases (as a health indicator) be qualified as health data. While this provision potentially allows for more effective bias mitigation, and simultaneously offers further clarification on the interaction of AI medical devices with the GDPR, it is still not crystal clear when the threshold of ‘appropriate safeguards’ is met. This legal uncertainty can be both to manufacturers of AI medical devices (risking high GDPR fines) and individuals (risking privacy violations because of possible abuse of the exemption or data breaches).^129^ Another issue arising from placing the post-market surveillance of biases with the provider of AI medical devices is that the provider is dependent on data from external parties, such as publicly available scientific reports, or adverse effects reported by health professionals or patients. The availability of this sensitive (health) data is also governed by the GDPR—meaning that the data are often high-level and do not always contain the information needed to assess bias for specific patients, which is necessary in case of ‘invisible biases’ such as ageism.

## VI. CONCLUSIONS AND THE WAY FORWARD

In conclusion, the existing MDR demonstrates significant shortcomings in addressing ageism in AI medical devices. These limitations primarily arise from the lack of guidance on bias assessment, clinical evaluation, and relations to other legislative frameworks, as well as limited transparency and public disclosure. The MDR’s broad scope and high-level requirements for software hinder its effectiveness in accommodating the unique characteristics of AI medical devices.

The new conformity requirements introduced by the AI Act offer potential solutions, but their effectiveness is contingent upon the yet-to-be-published content of conformity standards by EU standardization bodies and approval by the European Commission. Furthermore, while the proposal emphasizes the importance of bias reduction, it primarily focuses on biases in the underlying data and lacks provisions to address contextual biases related to stereotypes, prejudices, and unconscious biases. This limits its effectiveness in combating discrimination, particularly in the application phase of AI systems. There is however some ‘low-hanging fruit’ to pick—which still can be addressed in the trilogue-negotiations. The AI Act obligation to register AI systems in an AI database could be extended to health professionals, public access to clinical evaluation information in the EUDAMED database could be extended to Class IIa and IIb medical devices to increase transparency and linking the EUDAMED database with the AI database could provide a more comprehensive understanding of AI-based devices to guide medical decision-making.

These solutions do however not detract from the fact the key issue with the EU regulatory framework for AI medical devices is its narrow understanding of the challenges posed by bias in AI. Bias is regarded as an issue of *patient safety* and *product performance*. By framing the issue of bias in this manner, instead of from the perspective of *fundamental rights protection* and *health (in)equity*, the choice was made for the regulatory measures for bias mitigation to primarily address product performance thus focusing solely on the product *itself*, and not on the wider context in which the product is developed and used.

The AI cycle of health inequity shows that the issue of bias extends far beyond the product itself. A product safety approach to AI medical devices is insufficient for adequately mitigating biases, especially the more invisible bias of digital ageism, as part of the sources of age biases exist in the real world, external to the product. The current EU framework does address some of the key issues related to *technical* biases in AI medical devices by stipulating rules for performance and data quality but does not account for *contextual* biases, therefore neglecting an important part of the cycle of health inequity. A significant portion of digital ageism arises from how these systems are deployed, including considerations such as whether health professionals prescribe them to older patients and the level of health literacy involved.

Considering that AI is not merely a product but a complex system, the EU’s regulatory paradigm, primarily designed for product regulation, requires a comprehensive system approach to effectively regulate AI. A fundamental rights approach to AI MDR would center on the impact of the device on individuals (eg the health professional and the user) in every single phase of the lifecycle of the AI medical device, rather than the product itself. At the same time, it is important to recognize that the AI cycle of health inequity resulting from age—and other—biases in AI medical devices extends beyond *individual* health status and *individual* fundamental rights protection, and in fact reinforces persisting ageism (and other forms of discrimination) in society, eventually leading to health inequity.

The MDR demonstrates inadequate adaptation to the new reality of AI-based medical devices. The AI Act proposes some improvements but fails to adequately address the health-specific nature of AI medical devices. In light of these findings, it is imperative for policymakers, regulators, and stakeholders to recognize the limitations of existing regulations and work toward a comprehensive and tailored approach to addressing ageism in AI medical devices. This entails incorporating robust guidance, promoting transparency, addressing deployment practices, and establishing a health-specific legal framework for data governance. Only through such efforts can the potential of AI technology be harnessed to ensure equitable and effective healthcare for all age groups. Today, the AI Act is only a proposal: it is now in the hands of the Parliament and the Council to ensure health equity in the AI Act.

## Footnotes

1 Bertalan Meskó, Gergely Hetényi & Zsuzsanna Győrffy, *Will Artificial Intelligence Solve the Human Resource Crisis in Healthcare?*, 18 BMC Health Serv. Res. 545 (2018), https://doi.org/10.1186/s12913-018-3359-4 (accessed May 24, 2023).

2 Moonika Raja et al., *Telehealth and Digital Developments in Society That Persons 75 Years and Older in European Countries Have Been Part of: A Scoping Review*, 21 BMC Health Serv. Res. 1157 (2021), https://doi.org/10.1186/s12913-021-07154-0 (accessed May 23, 2023).

3 Examples taken from the European Commission’s EUDAMED database consulted on May 12, 2023.

4
European Union Agency for Fundamental Rights, Bias in Algorithms—Artificial Intelligence and Discrimination (2022).

5 Justyna Stypinska, *AI Ageism: A Critical Roadmap for Studying Age Discrimination and Exclusion in Digitalized Societies*, AI Soc. (2022), https://doi.org/10.1007/s00146-022-01553-5 (accessed Apr. 21, 2023).

6 European Union Directorate General for Communication. Special Eurobarometer 493: discrimination in the EU. Brussels: European Commission; 2019.

7 Robert N. Butler, *Age-Ism: Another Form of Bigotry*, 9 The Gerontologist 243 (1969), https://doi.org/10.1093/geront/9.4_Part_1.243 (accessed Apr. 26, 2023).

8 Charlene H. Chu et al., *Digital Ageism: Challenges and Opportunities in Artificial Intelligence for Older Adults*, 62 The Gerontologist 947 (2022), https://doi.org/10.1093/geront/gnab167 (accessed Apr. 12, 2023).

9 Justyna Stypinska, *AI Ageism: A Critical Roadmap for Studying Age Discrimination and Exclusion in Digitalized Societies*, 38 Ai & Society 665 (2023).

10
World Health Organization, *Ageism in Artificial Intelligence for Health: WHO Policy Brief* (2022).

11
World Health Organization, *Global Report on Ageism* (2021).

12
*Id.*

13 Andrea Rosales & Mireia Fernández-Ardèvol, *Structural Ageism in Big Data Approaches*, 40 Nord. Rev. 51 (2019), https://sciendo.com/article/10.2478/nor-2019-0013?tab=ilustracje-i-tabele (accessed Apr. 21, 2023).

14 Also see on ‘cascading effects’ of medical AI: David Leslie et al., *Does ‘AI’ Stand for Augmenting Inequality in the Era of Covid-19 Healthcare?*, 372 BMJ n304 (2021), https://www.bmj.com/content/372/bmj.n304 (accessed May 4, 2023); World Health Organization, *Health Inequities and Their Causes* (2018), https://www.who.int/news-room/facts-in-pictures/detail/health-inequities-and-their-causes (accessed Oct. 11, 2023).

15 Sharon K. Inouye, *Creating an Anti-Ageist Healthcare System to Improve Care for Our Current and Future Selves*, 1 Nat. Aging 150 (2021), https://www.nature.com/articles/s43587-020-00004-4 (accessed Apr. 25, 2023).

16 Cynthia J. Sieck and others, *Digital Inclusion as a Social Determinant of Health*, 4 Digital Medicine 1 (2021).

17 Council of Europe, European Court of Human Rights and European Union Agency for Fundamental Rights (EU body or agency), *Handbook on European Non-Discrimination Law: 2018 Edition* (Publications Office of the European Union 2018), p. 20–23.

18 Regulation (EU) 2017/745 on Medical Devices.

19 Article 20 MDR.

20 Proposal for a Regulation of the European Parliament and of the Council laying down Harmonised Rules on Artificial Intelligence (Artificial Intelligence Act) and amending certain Union Legislative Acts, Com/2021/206 Final.

21 Gabriele Mazzini & Salvatore Scalzo, *The Proposal for the Artificial Intelligence Act: Considerations around Some Key Concepts* (2022), https://papers.ssrn.com/abstract=4098809 (accessed May 30, 2023).

22 See AI Act, Annex II, Section A under 11.

23 Butler, *supra* note 7.

24
World Health Organization, *supra* note 11.

25 Daisy Neal et al., *Is There Evidence of Age Bias in Breast Cancer Health Care Professionals’ Treatment of Older Patients?*, 48 Eur. J. Surg. Oncol. 2401 (2022), https://www.sciencedirect.com/science/article/pii/S0748798322005455 (accessed Apr. 26, 2023); Lisa J. M. Caldon et al., *What Influences Clinicians’ Operative Preferences for Women with Breast Cancer? An Application of the Discrete Choice Experiment*, 43 Eur. J. Cancer 1662 (2007), https://www.sciencedirect.com/science/article/pii/S0959804907003486 (accessed Apr. 27, 2023); Neal et al.; A. K. Madan, S. Aliabadi-Wahle & D. J. Beech, *Ageism in Medical Students’ Treatment Recommendations: The Example of Breast-Conserving Procedures*, 76 Acad. Med. J. Assoc. Am. Med. Coll. 282 (2001).

26 Inouye, *supra* note 18.

27 E.-Shien Chang et al., *Global Reach of Ageism on Older Persons’ Health: A Systematic Review*, 15 PLOS ONE e0220857 (2020), https://journals.plos.org/plosone/article?id=10.1371/journal.pone.0220857 (accessed Apr. 25, 2023).

28 Rita Banzi et al., *Older Patients Are Still Under-Represented in Clinical Trials of Alzheimer’s Disease*, 8 Alzheimers Res. Ther. 32 (2016), https://doi.org/10.1186/s13195-016-0201-2 (accessed May 3, 2023).

29 Chang et al., *supra* note 30.

30 PhD Gutterman, *Ageism and Older Women* (2023), https://papers.ssrn.com/abstract=4412525 (accessed May 3, 2023).

31 E-Shien Chang and others, ‘*Global Reach of Ageism on Older Persons’ Health: A Systematic Review*’ (2020) 15 PLOS ONE e0220857.

32
*Supra* note 10.

33 Kanlun Wang, Lina Zhou and Dongsong Zhang, ‘*Biometrics-Based Mobile User Authentication for the Elderly: Accessibility, Performance, and Method Design*’, 1 Int. J. Hum. Comput. Int. (2023).

34
Giovanni Zaccaroni, Equality and Non-Discrimination in the EU: The Foundations of the EU Legal Order (2021).

35 Council Directive 2000/78/EC of 27 Nov. 2000 establishing a general framework for equal treatment in employment and occupation, OJ L 303, 02/12/2000 P. 0016–0022.

36 Proposal for a Council Directive on implementing the principle of equal treatment between persons irrespective of religion or belief, disability, age or sexual orientation {SEC(2008) 2180} {SEC(2008) 2181} /* COM/2008/0426 final—CNS 2008/0140 */.

37 White Paper on Artificial Intelligence: A European approach to excellence and trust, Brussels, 19.2.2020 COM(2020) 65 final.

38 Sandra Wachter, Brent Mittelstadt & Chris Russell, *Why Fairness Cannot Be Automated: Bridging the Gap between EU Non-Discrimination Law and AI*, 41 Comput. Law Secur. Rev. 105567 (2021), https://www.sciencedirect.com/science/article/pii/S0267364921000406 (accessed May 21, 2023).

39 Hannah van Kolfschooten & Carmel Shachar, *The Council of Europe's AI Convention (2023–2024): Promises and pitfalls for health protection*, 138 Health Policy 104935 (2023), https://www.sciencedirect.com/science/article/pii/S0168851023002208 (accessed Nov 8, 2023).

40 Reva Schwartz et al., *Towards a Standard for Identifying and Managing Bias in Artificial Intelligence*, NIST (2022), https://www.nist.gov/publications/towards-standard-identifying-and-managing-bias-artificial-intelligence (accessed Apr. 19, 2023).

41
*Id.*

42 The Lancet Digital Health, *Digital Technologies: A New Determinant of Health*, 3 Lancet Digit. Health e684 (2021), https://www.thelancet.com/journals/landig/article/PIIS2589-7500(21)00238-7/fulltext (accessed May 4, 2023).

43 Safiya Richardson et al., *A Framework for Digital Health Equity*, 5 Npj Digit. Med. 1 (2022), https://www.nature.com/articles/s41746-022-00663-0 (accessed May 4, 2023).

44
*Id.*

45 Eurostat, ISOC_CI_AC_I, last data update: Mar. 7, 2023.

46 Sandra Florisson et al., *Are Older Adults Insufficiently Included in Clinical Trials?—An Umbrella Review*, 128 Basic Clin. Pharmacol. Toxicol. 213 (2021), https://onlinelibrary.wiley.com/doi/abs/10.1111/bcpt.13536 (accessed Apr. 24, 2023).

47 Trishan Panch, Heather Mattie & Leo Anthony Celi, *The ‘Inconvenient Truth’ about AI in Healthcare*, 2 Npj Digit. Med. 1 (2019), https://www.nature.com/articles/s41746-019-0155-4 (accessed Feb. 20, 2023).

48 Neal et al., *supra* note 28.

49 Inouye, *supra* note 18.

50 Banzi et al., *supra* note 31.

51 W. Nicholson II Price, *Medical AI and Contextual Bias*, 33 Harv. J. Law Technol. Harv. JOLT 65 (2019), https://heinonline.org/HOL/P?h=hein.journals/hjlt33&i=71 (accessed Jan. 27, 2023).

52 Angela Kydd & Anne Fleming, *Ageism and Age Discrimination in Health Care: Fact or Fiction? A Narrative Review of the Literature*, 81 Maturitas 432 (2015), https://www.sciencedirect.com/science/article/pii/S0378512215006738 (accessed Apr. 26, 2023).

53 Barbara Mikołajczyk, *Universal Human Rights Instruments and Digital Literacy of Older Persons*, 27 Int. J. Hum. Rights 403 (2023), https://doi.org/10.1080/13642987.2022.2131772 (accessed May 16, 2023).

54 Eurostat, ISOC_CI_IFP_IU, last update: Mar. 7, 2023.

55 Inouye, *supra* note 18.

56 Leslie et al., *supra* note 14.

57 Gutterman, *supra* note 33.

58 Article 2 TEU.

59 Article 168(1) TFEU.

60 Article 168(4)(c) TFEU.

61 Article 114 TFEU.

62 Recital 1, MDR.

63 Article 2(1), MDR. NB: Also Annex XVI-devices qualify as medical devices according to Article 1(2) MDR.

64 Timo Minssen, Marc Mimler & Vivian Mak, *When Does Stand-Alone Software Qualify as a Medical Device in the European Union?—The Cjeu’s Decision in Snitem and What It Implies for the Next Generation of Medical Devices*, 28 Med. Law Rev. 615 (2020).

65
*Id.*

66 MDCG 2019–11, Guidance on Qualification and Classification of Software in Regulation (EU) 2017/745—MDR and Regulation (EU) 2017/746—IVDR, Oct. 2019.

67 MDR Annex VIII, section 6.3, Rule 11).

68 R. Beckers, Z. Kwade & F. Zanca, *The EU Medical Device Regulation: Implications for Artificial Intelligence-Based Medical Device Software in Medical Physics*, 83 Phys. Med. 1 (2021). Kerstin N Vokinger, Thomas J Hwang and Aaron S Kesselheim, *Lifecycle Regulation and Evaluation of Artificial Intelligence and Machine Learning-Based Medical Devices* in The Future of Medical Device Regulation: Innovation and Protection (Cambridge University Press, Carmel Shachar et al. eds., 2022).

69 MDR, Annex I, Chapter 1.

70 Article 32 MDR.

71 Article 87 (1) MDR.

72 MDR, Annex XV, Chapter II Clinical Investigations, 3.6.4.

73 Article 62 MDR.

74 See the European Commision’s ‘Summary of references of harmonised standards published in the Official Journal—Regulation (EU) 2017/745’, May 17, 2022.

75 MDCG, Summary of safety and clinical performance: A guide for manufacturers and notified bodies, Mar. 2022, 2019-9 Rev. 1.

76 See MDR, Annex XV.

77 COCIR, Artificial Intelligence in Medical Device Legislation, Sept. 2020, p. 20–21.

78
*What the radiologist should know about artificial intelligence—an ESR white paper*, 10 Insights Imaging 44 (2019), https://www.ncbi.nlm.nih.gov/pmc/articles/PMC6449411/ (accessed May 19, 2023).

79 Reference databases are online collections of data on a specific topic, for example genetic data or all performance data from studies on a specific disease. Examples of common databases for literature review are Embase, MEDLINE, and Biosis. Examples of common reference databases are the Orphadata platform and UniProt.

80 Janos Meszaros, Marcelo Corrales Compagnucci and Timo Minssen, *The Interaction of the Medical Device Regulation and the GDPR: Do European Rules on Privacy and Scientific Research Impair the Safety and Performance of AI Medical Devices?* in The Future of Medical Device Regulation: Innovation and Protection (Carmel Shachar et al. eds., 2022).

81 Article 109 and 110 MDR.

82 Alan G. Fraser et al., *The Need for Transparency of Clinical Evidence for Medical Devices in Europe*, 392 Lancet Lond. Engl. 521 (2018).

83 Stephanie R. Fox-Rawlings et al., *Diversity in Medical Device Clinical Trials: Do We Know What Works for Which Patients?*, 96 Milbank Q. 499 (2018).

84 Boris Babic et al., *Algorithms on Regulatory Lockdown in Medicine*, 366 Science 1202 (2019), https://www.science.org/doi/full/10.1126/science.aay9547 (accessed May 21, 2023).

85 Matthew DeCamp & Charlotta Lindvall, *Latent Bias and the Implementation of Artificial Intelligence in Medicine*, 27 J. Am. Med. Inform. Assoc. JAMIA 2020 (2020), https://www.ncbi.nlm.nih.gov/pmc/articles/PMC7727353/ (accessed May 21, 2023).

86 MDR, Annex IX, Article 4.10.

87 Babic et al., *supra* note 86.

88 Thomas P. Quinn and others, *Trust and Medical AI: The Challenges We Face and the Expertise Needed to Overcome Them*, 28 Journal of the American Medical Informatics Association: JAMIA 890 (2020).

89 The MDCG drafts technical recommendations on matters relating to notified bodies and conformity assessment and provides advice to the Commission on the MDR implementation. Also see the previous MEDDEV guideline 2.7/1 revision 4 (June 2016) on clinical evaluation under the previous Medical Devices Directives.

90 MDCG 2019-11, Guidance on Qualification and Classification of Software in Regulation (EU) 2017/745—MDR and Regulation (EU) 2017/746—IVDR, Oct. 2019.

91 Recital 83, MDR.

92 Alan G. Fraser et al., *Artificial Intelligence in Medical Device Software and High-Risk Medical Devices*—*A Review of Definitions, Expert Recommendations and Regulatory Initiatives*, 0 Expert Rev. Med. Devices 1 (2023), https://doi.org/10.1080/17434440.2023.2184685 (accessed May 18, 2023).

93
*Id.*

94 FDA, *Marketing Submission Recommendations for a Predetermined Change Control Plan for Artificial Intelligence/Machine Learning (AI/ML)-Enabled Device Software Functions: Draft Guidance for Industry and Food and Drug Administration Staff*, 3 Apr. 2023; FDA, *Artificial Intelligence/Machine Learning (AI/ML)-Based Software as a Medical Device (SaMD) Action Plan*, Jan. 2021.

95
Scott L. Greer et al., Everything You Always Wanted to Know about European Union Health Policies but Were Afraid to Ask (2022).

96 Marco Almada & Nicolas Petit, *The EU AI Act: Between Product Safety and Fundamental Rights*, (2022), https://papers.ssrn.com/abstract=4308072 (accessed Apr. 4, 2023).

97 Recital 1, AI Act proposal (COM/2021/206 final).

98 Annex III, AI Act proposal (COM/2021/206 final).

99 Michael Veale & Frederik Zuiderveen Borgesius, *Demystifying the Draft EU Artificial Intelligence Act*, 22 Comput. Law Rev. Int. 97 (2021), http://arxiv.org/abs/2107.03721 (accessed July 4, 2022).

100 Article 294, Treaty on the Functioning of the European Union (TFEU).

101 Article 6 and Annex II number 11 AI Act proposal (COM/2021/206 final).

102 Hannah van Kolfschooten, *EU Regulation of Artificial Intelligence: Challenges for Patients’ Rights* 59 Common Market Law Review (2022).

103 See the documents comprising the ‘European approach to AI’ here: https://digital-strategy.ec.europa.eu/en/policies/european-approach-artificial-intelligence (accessed 10 June, 2023).

104 Recital 3, AI Act Proposal (COM/2021/206 final).

105 See Recital 15 and 16, AI Act Proposal (COM/2021/206 final).

106 NB: In the negotiations within the EU Parliament, a new article on taking measures for digital literacy is proposed.

107 Article 7(2)(f), AI Act proposal (COM/2021/206 final).

108 Recital 16, AI Act proposal (COM/2021/206 final).

109 Recital 33, AI Act proposal (COM/2021/206 final).

110 Recital 36 and 37, AI Act proposal (COM/2021/206 final) (accessed 10 June, 2023).

111 Such as for example the GDPR.

112 Article 10, AI Act proposal (COM/2021/206 final).

113 Article 13, AI Act proposal (COM/2021/206 final).

114 Article 14, AI Act proposal (COM/2021/206 final).

115 Proposal for a REGULATION OF THE EUROPEAN PARLIAMENT AND OF THE COUNCIL on the European Health Data Space, Strasbourg, 3.5.2022, COM(2022) 197 final, 2022/0140(COD).

116 Article 10(3) and 10(4), AI Act proposal (COM/2021/206 final).

117 Article 10(5) and 10(6), AI Act proposal (COM/2021/206 final).

118 Article 11 and 12, AI Act proposal (COM/2021/206 final).

119 Explanatory Memorandum, §1.1, AI Act proposal (COM/2021/206 final).

120
World Health Organization, *supra* note 10.

121 Articles 13, 14, and 52, AI Act proposal (COM/2021/206 final).

122 Article 60 and 61, AI Act proposal (COM/2021/206 final).

123 See Article 3(1), point 11 and 13, AI Act proposal (COM/2021/206 final).

124 Almada and Petit, *supra* note 98.

125 Sara Gerke et al., *The Need for a System View to Regulate Artificial Intelligence/Machine Learning-Based Software as Medical Device*, 3 Npj Digit. Med. 1 (2020), https://www.nature.com/articles/s41746-020-0262-2 (accessed May 18, 2023).

126 Mazzini and Scalzo, *supra* note 24.

127 Article 43(4), AI Act proposal (COM/2021/206 final).

128 Article 10(5), AI Act proposal (COM/2021/206 final).

129 Marvin van Bekkum & Frederik Zuiderveen Borgesius, *Using Sensitive Data to Prevent Discrimination by Artificial Intelligence: Does the GDPR Need a New Exception?*, 48 Comput. Law Secur. Rev. 105770 (2023), https://www.sciencedirect.com/science/article/pii/S0267364922001133 (accessed Nov. 29, 2022).

